# Supplementary material for: Statistical approaches for service delivery differentials as assessed through a composite indicator: Application to Ugandan local governments
Source: PLoS One. 2025 Dec 11;20(12):e0338264. doi: 10.1371/journal.pone.0338264 (PMC12698004; doi:10.1371/journal.pone.0338264)
Supplement: S1 Table — (DOCX) [file pone.0338264.s001.docx]

**S1 Table:** Elementary indicators used

| **No** | **Indicator** | **Var. name** | **Unit** | **Utopian Value** | **Dystopian Value** | **Utopian Type** | **Dystopian Type** | **Service-delivery standard** | **Frequency of Production** | **Source** | **Level in the Theoretical Framework** |  |
| --- | --- | --- | --- | --- | --- | --- | --- | --- | --- | --- | --- | --- |
|  | **Health Dimension** | | | | | | | | | | | |
| 1 | Percentage of approved posts filled | **h1** | Percent | 86 | 39.8 | Ministry of Health Strategic Plan target | Least reported |  | Annual | Sector Performance Report | Input |  |
| 2 | Deliveries in health facilities | **h2** | Percent | 100 | 33.2 | Maximum possible | Least reported |  | Annual | Sector Performance Report | Process |  |
| 3 | Health Centre III: Sub-county ratio | **h3** | Ratio | 1 | 0 | Uganda Health Minimum Package | Least reported | Every sub-county should have at least a government health centre III | Annual | Master List of Health Institutions | Input |  |
| 4 | Number of registered health centres | **h4** | Number | 34166 | 1211 | Maximum reported | Least reported |  | Annual | Sector Performance Report | Input |  |
|  | **Water Dimension** | | | | | | | | | | | |
| 5 | Rural access to water | **w1** | Percent | 95 | 27 | Maximum reported | Least reported |  | Annual | Programme performance report | Outcome |  |
| 6 | Urban access to water | **w2** | Percent | 95 | 0 | Maximum reported | Least reported |  | Annual | Programme performance report | Outcome |  |
| 7 | Rural water systems functional | **w3** | Percent | 100 | 0 | NDP Target | Least possible |  | Annual | Programme performance report | Input |  |
| 8 | Urban systems functional | **w4** | Percent | 100 | 0 | Maximum reported | Least 0 |  | Annual | Programme performance report | Input |  |
| 9 | Water-use committees functional | **w5** | Percent | 100 | 0 | Maximum reported | Least reported |  | Annual | Programme performance report | Process |  |
| 10 | Villages with an improved water source | **w6** | Percent | 100 | 0 | Maximum reported | Least reported |  | Annual | Programme performance report | Outcome |  |
| 11 | Handwashing coverage | **w7** | Percent | 97 | 0 | Maximum reported | Least reported |  | Annual | Programme performance report | Outcome |  |
| 12 | Sanitation coverage | **w8** | Percent | 99.8 | 8.8 | Maximum reported | Least reported |  | Annual | Programme performance report | Outcome |  |
|  | **Education Dimension** | | | | | | | | | | | |
| 13 | Pupil: Teacher Ratio | **e1** | Ratio | 40 | 133 | Global Partnership for Education service delivery standard | Least reported | The global standard is 1:40 | Administrative Data | Derived indicator | Input |  |
| 14 | Student: Teacher Ratio | **e2** | Ratio | 40 | 53 | Maximum reported | Least reported |  | Administrative Data | Derived indicator | Input |  |
| 15 | Number of pre-primary schools | **e3** | Number | 4261 | 3 | Maximum reported | Least reported |  | Administrative Data | Master List of Education Institutions | Input |  |
| 16 | Number of certificate awarding institutions | **e4** | Number | 127 | 0 | Maximum reported | Least reported |  | Administrative Data | Master List of Education Institutions | Input |  |
| 17 | Primary school: parish ratio | **e5** | Ratio | 1 | 0.4 | Ministry of Education target | Least reported | Every parish should have at least one government primary school | Administrative Data | Derived indicator | Input |  |
| 18 | Secondary school: LLG ratio | **e6** | Ratio | 1 | 0.1 | Ministry of Education target | Least reported | Every sub-county should have at least one secondary school | Administrative Data | Derived indicator | Input |  |
| 19 | Primary Leaving Examination pass rate | **e7** | Percent | 100 | 59 | Maximum possible | Least reported |  | Annual | UNEB Report | Outcome |  |
| 20 | Uganda Certificate of Education pass rate | **e8** | Percent | 100 | 49 | Maximum possible | Least reported |  | Annual | UNEB Report | Outcome |  |
