# Supplementary material for: Statistical approaches for service delivery differentials as assessed through a composite indicator: Application to Ugandan local governments
Source: PLoS One. 2025 Dec 11;20(12):e0338264. doi: 10.1371/journal.pone.0338264 (PMC12698004; doi:10.1371/journal.pone.0338264)
Supplement: S2 Table — (DOCX) [file pone.0338264.s002.docx]

**S2 Table:** Cross frequencies among categorical variables

|  |  | **Has MC or City** | |  | **Is PRDP district** | |  | **Is refugee hosting** | |
| --- | --- | --- | --- | --- | --- | --- | --- | --- | --- |
|  |  | **No** | **Yes** |  | **No** | **Yes** |  | **No** | **Yes** |
| Disaster risk | No | 0.54 | 0.14 |  | 0.49 | 0.19 |  | 0.06 | 0.62 |
|  | Yes | 0.22 | 0.10 |  | 0.24 | 0.08 |  | 0.02 | 0.3 |
|  |  |  |  |  |  |  |  |  |  |
|  |  | **Has MC or City** | |  | **Is refugee hosting** | |  |  |  |
|  |  | **No** | **Yes** |  | **No** | **Yes** |  |  |  |
| Is a PRDP district | No | 0.54 | 0.18 |  | 0.06 | 0.67 |  |  |  |
|  | Yes | 0.22 | 0.06 |  | 0.02 | 0.26 |  |  |  |
