# Supplementary material for: Statistical approaches for service delivery differentials as assessed through a composite indicator: Application to Ugandan local governments
Source: PLoS One. 2025 Dec 11;20(12):e0338264. doi: 10.1371/journal.pone.0338264 (PMC12698004; doi:10.1371/journal.pone.0338264)
Supplement: S3 Table — (DOCX) [file pone.0338264.s003.docx]

**S3 Table:** Correlation matrix for the continuous variables

|  | **popn_2021** | **land_area** | **sub_cty** | **town_cl** | **parish** | **age_2022** | **distance_kla** | **local_revenue** | **centgovt_fund** | **centgovt_other** | **donor_fund** | **exp_3sectors** |
| --- | --- | --- | --- | --- | --- | --- | --- | --- | --- | --- | --- | --- |
| popn_2021 | 1.00 | -0.01 | 0.15 | 0.42 | 0.55 | 0.36 | -0.34 | 0.16 | 0.61 | 0.06 | 0.00 | 0.52 |
| land_area | -0.01 | 1.00 | 0.04 | -0.06 | -0.08 | 0.07 | 0.25 | 0.16 | -0.01 | 0.09 | 0.15 | -0.16 |
| sub_cty | 0.15 | 0.04 | 1.00 | 0.36 | 0.43 | 0.00 | 0.12 | -0.14 | 0.25 | -0.12 | -0.11 | 0.29 |
| town_cl | 0.42 | -0.06 | 0.36 | 1.00 | 0.37 | 0.14 | -0.02 | 0.06 | 0.43 | -0.18 | -0.17 | 0.45 |
| parish | 0.55 | -0.08 | 0.43 | 0.37 | 1.00 | 0.43 | -0.10 | 0.18 | 0.44 | -0.13 | -0.09 | 0.36 |
| age_2022 | 0.36 | 0.07 | 0.00 | 0.14 | 0.43 | 1.00 | -0.02 | 0.24 | 0.35 | -0.13 | -0.07 | 0.13 |
| distance_kla | -0.34 | 0.25 | 0.12 | -0.02 | -0.10 | -0.02 | 1.00 | -0.07 | -0.15 | 0.20 | 0.12 | -0.19 |
| local_revenue | 0.16 | 0.16 | -0.14 | 0.06 | 0.18 | 0.24 | -0.07 | 1.00 | 0.18 | 0.09 | -0.03 | 0.15 |
| centgovt_fund | 0.61 | -0.01 | 0.25 | 0.43 | 0.44 | 0.35 | -0.15 | 0.18 | 1.00 | -0.02 | -0.10 | **0.86** |
| centgovt_other | 0.06 | 0.09 | -0.12 | -0.18 | -0.13 | -0.13 | 0.20 | 0.09 | -0.02 | 1.00 | 0.43 | 0.11 |
| donor_fund | 0.00 | 0.15 | -0.11 | -0.17 | -0.09 | -0.07 | 0.12 | -0.03 | -0.10 | 0.43 | 1.00 | **-0.03** |
| exp_3sectors | 0.52 | -0.16 | 0.29 | 0.45 | 0.36 | 0.13 | -0.19 | 0.15 | **0.86** | 0.11 | **-0.03** | 1.00 |
