# Supplementary material for: Statistical approaches for service delivery differentials as assessed through a composite indicator: Application to Ugandan local governments
Source: PLoS One. 2025 Dec 11;20(12):e0338264. doi: 10.1371/journal.pone.0338264 (PMC12698004; doi:10.1371/journal.pone.0338264)
Supplement: S4 Table — (DOCX) [file pone.0338264.s004.docx]

**S4 Table:** Output of application of GAM

|  |  |  | **ANOVA for parametric effect** | | | | **ANOVA for non-parametric effects** | | |
| --- | --- | --- | --- | --- | --- | --- | --- | --- | --- |
| **Variable** | **Coefficients** | **EDF** | **Sum Sq** | **Mean Sq** | **F value** | **Pr(>F)** | **F value** | **Pr(>F)** | **EDF** |
| Intercept | 3.9518e-01 |  |  |  |  |  |  |  |  |
| Is_refugee hosting district | 1.0816e-02 | 1 | 0.0019 | 0.0019 | 0.8001 | 0.3730 |  |  |  |
| s(sub_cty, df = 6) | -4.7618e-03 | 1 | 0.0254 | 0.0254 | 10.7744 | 0.0014** | 4.8111 | 0.0005 *** | 6 |
| town_cl | 3.3934e-03 | 1 | 0.0346 | 0.0346 | 14.6651 | 0.0002 *** |  |  |  |
| s(Centgovt_fund, df = 6) | 3.2358e-05 | 1 | 0.0677 | 0.0677 | 28.6604 | 4.829e-07 *** | 2.8647 | 0.0180* | 6 |
| Residuals |  |  | 0.2573 | 0.002361 |  |  |  |  |  |
|  |  |  | AIC: -382.1369 | | | |  |  |  |

*Significance codes ***0.001, **0.01, *0.05*
