# Supplementary material for: Statistical approaches for service delivery differentials as assessed through a composite indicator: Application to Ugandan local governments
Source: PLoS One. 2025 Dec 11;20(12):e0338264. doi: 10.1371/journal.pone.0338264 (PMC12698004; doi:10.1371/journal.pone.0338264)
Supplement: S1 Fig — (DOCX) [file pone.0338264.s005.docx]

*

*

**S1 Figure:** OOB error rate for RF regression Vs number of trees grown
