# Supplementary material for: Statistical approaches for service delivery differentials as assessed through a composite indicator: Application to Ugandan local governments
Source: PLoS One. 2025 Dec 11;20(12):e0338264. doi: 10.1371/journal.pone.0338264 (PMC12698004; doi:10.1371/journal.pone.0338264)
Supplement: S2 Fig — (DOCX) [file pone.0338264.s006.docx]

**S2 Figure:** Variable importance plot returned by RF
