# Supplementary material for: Statistical approaches for service delivery differentials as assessed through a composite indicator: Application to Ugandan local governments
Source: PLoS One. 2025 Dec 11;20(12):e0338264. doi: 10.1371/journal.pone.0338264 (PMC12698004; doi:10.1371/journal.pone.0338264)
Supplement: Attachment — Submitted filename: Recommendation.pdf [file pone.0338264.s008.pdf]

This study focuses on service delivery by local governments in Uganda. It constructs a composite indicator and applies multiple models (e.g., Beta regression, random forest) to analyze influencing factors. The research design aligns with the practical needs of public management, and the data processing and methodological application demonstrate basic rigor, providing a practical reference for the assessment of local governance in Uganda. However, there is room for improvement in terms of theoretical linkage, methodological details, result interpretation, and policy extension. Further refinement is needed to meet the requirements of PLOS ONE for research quality and contribution.

Specific Suggestions：

Major Comments：

1.The construction of the composite indicator refers to the OECD process but fails to link with classic theories of public service delivery. The selection of indicators and weighting schemes lacks in-depth integration with theoretical goals such as “fairness and efficiency” in service delivery.

Suggestion: Supplement the elaboration of the theoretical framework. Explain how the indicators correspond to the dimensions of “service inputs, processes, and outcomes.” Combine public service assessment theories to justify the rationality of the weighting schemes, providing a stronger theoretical basis for method selection.

2.The literature primarily focuses on domestic policies in Uganda, with insufficient references to similar international studies. It is difficult to highlight the theoretical contribution of this research in the field of “local government service governance.”

Suggestion: Supplement comparisons with international literature and analyze the uniqueness of the Ugandan case.

3.The analysis of the impact of key variables merely stays at the description of “significantly positive/negative correlation” and fails to explain the mechanisms in combination with the actual governance context of Uganda. For categorical variables, only frequency statistics are provided, without in-depth analysis of their impact paths on service delivery.

Suggestion: Explain the influence mechanisms of variables by combining details of the operation of local governments in Uganda.

4.The conclusion mentions “providing references for policymakers” but does not propose specific plans based on key findings.

Suggestion: Based on the model results, refine policy recommendations.

5.The discussion of limitations only generally mentions that “indicator dimensions can be expanded and variables can be supplemented,” failing to address core deficiencies. The planning for future research directions is relatively vague and lacks specificity.

Suggestion: Supplement the analysis of key limitations, clarify improvement paths, and demonstrate the sustainability of the research.

Minor Comments：

1.For Figure 1, adding textual annotations for key steps to lower the comprehension barrier for readers.

2.Strengthen the correlation analysis between Figure 2 and Table 2. For example, supplement the discussion on “the impact of variables with high skewness/kurtosis on the distribution pattern of the composite indicator” and provide further explanations.

3.For the“*”marking “Leptokurtic variable” in Table 2, clearly define the judgment criteria for kurtosis abnormalities in the table notes or main text.

4.For the sub-figure titles in Figure 2, supplement explanations of the data period and analysis unit to enhance the self-explanatory nature of the figures.

5.There are grammatical issues in the sentences on Line 306 and Line 595; revisions are required.

6.Further optimize the format and arrangement of figures and tables (e.g., Table 8) for better presentation.
