# Supplementary material for: Statistical approaches for service delivery differentials as assessed through a composite indicator: Application to Ugandan local governments
Source: PLoS One. 2025 Dec 11;20(12):e0338264. doi: 10.1371/journal.pone.0338264 (PMC12698004; doi:10.1371/journal.pone.0338264)
Supplement: Attachment — Submitted filename: Recommendations.pdf [file pone.0338264.s009.pdf]

1.The study mentions that the construction of the composite indicator refers to the OECD process and integrates the "World Bank's Public Administration Production Function" and the "Public Service Assessment Methodology". However, it fails to clearly specify how these theories specifically guide indicator design: for instance, the corresponding relationship between the "service input-process-outcome" dimensions and the "student-teacher ratio" (input) in the education sector, "medical institution coverage rate" (process) in the health sector, and "safe drinking water access rate" (outcome) in the water sector. Although indicators are listed in Table A.1, their connection to theoretical dimensions is not established. It is recommended to supplement text to directly link core indicators to the dimensions in the theoretical framework, thereby enhancing the clarity of theoretical support through such connections.

2.The analysis of the impact paths of categorical variables is insufficient. Regarding the variable "whether a district is a refugee-hosting area", the study only mentions that "refugee-hosting areas have slightly higher service delivery scores" but fails to decompose the underlying mechanism: whether it is the direct effect of supplementary funding from UNHCR or the driven service demand caused by the concentration of refugees. Further analysis is needed by incorporating the interaction effects between variables.

3.The discussion section can be deepened. For example, while the study mentions that "indicator dimensions can be expanded", it can supplement one specific potential small dimension. This revision does not question the completeness of the existing indicator system; it only makes the discussion on limitations more concrete and avoids excessive generality. It is recommended to separate the conclusion and discussion sections, with reference to the "Research Articles typically consist of the following headings" on the PLOS ONE official website.

4.In "by (47)" at Line 371, the author’s name is missing after "by". In English academic writing, when citing "a methodology proposed by a certain author", it is necessary to clearly state "by [Author Name] (47)". Simply writing "by (47)" will result in ambiguity regarding the citation object.

5.In the sentence "were incorporate using distance-to-reference point data normalization" at Line 382, the verb form is incorrect; "incorporate" should be changed to "incorporated" (passive voice requires the past participle form).

6.The word order of "on average" and "slightly" at Line 740 can be optimized to avoid semantic redundancy.

7.There are errors in the citation format. Please refer to the latest published papers and the requirements on the official website for revision.

8.There are issues with the reference format. Please refer to the latest published papers and the requirements on the official website for correction.

9.Most of the literatures cited in the study are from 2021 or earlier, and relevant studies on service delivery of Ugandan local governments from 2022 to 2024 have not been included.

10.The paragraph format still needs adjustment, particularly regarding issues with first-line indentation.
