# Supplementary material for: Statistical approaches for service delivery differentials as assessed through a composite indicator: Application to Ugandan local governments
Source: PLoS One. 2025 Dec 11;20(12):e0338264. doi: 10.1371/journal.pone.0338264 (PMC12698004; doi:10.1371/journal.pone.0338264)
Supplement: Attachment — Submitted filename: RebuttalLetter.docx [file pone.0338264.s010.docx]

**How Comments Have Been Addressed**

1. Please add a choropleth map of your variable

Thank you. As per your policy and advise, the map has been removed due to the inability to obtain permission from the original copyright holder.

2. The methods section can be shortened. You do not need to describe in detail regression, gam models, random forests and beta regression

Thank you. Some formulas have been removed (lines 260-272, and 201-210) and details summarized.

3. Note that gam models are SEMIPARAMETRIC.

Thank you. This has been corrected throughout the article.

4. Separate discussion and conclusion, and include a separate section on limitations.

Thank you. This has been addressed. The suggested limitations on data availability and dynamic nature of service performance have been included.

5. The study mentions that the construction of the composite indicator refers to the OECD process and integrates the "World Bank's Public Administration Production Function" and the "Public Service Assessment Methodology". However, it fails to clearly specify how these theories specifically guide indicator design: for instance, the corresponding relationship between the "service input-process-outcome" dimensions and the "student-teacher ratio" (input) in the education sector, "medical institution coverage rate" (process) in the health sector, and "safe drinking water access rate" (outcome) in the water sector. Although indicators are listed in Table A.1, their connection to theoretical dimensions is not established. It is recommended to supplement text to directly link core indicators to the dimensions in the theoretical framework, thereby enhancing the clarity of theoretical support through such connections.

Thank you. Lines 345-392 have been revised to show how the theories informed the theoretical framework ultimately informing the selection of elementary indicators and subsequent analyses. Table A.1 has been expanded to include a detailed explanation of the elementary indicators, particularly their linkage with the results chain/theoretical framework.

6. The literature primarily focuses on domestic policies in Uganda, with insufficient references to similar international studies. It is difficult to highlight the theoretical contribution of this research in the field of “local government service governance.” Suggestion: Supplement comparisons with international literature and analyze the uniqueness of the Ugandan case.

Thank you. This was addressed in lines 82-97. It shows that the Ugandan case builds a composite indicator, but also performs service delivery differentials across local governments

7. The analysis of the impact of key variables merely stays at the description of “significantly positive/negative correlation” and fails to explain the mechanisms in combination with the actual governance context of Uganda. For categorical variables, only frequency statistics are provided, without in-depth analysis of their impact paths on service delivery. Suggestion: Explain the influence mechanisms of variables by combining details of the operation of local governments in Uganda.

Thank you. This has been addressed in Lines 630 to 649 for beta regression. In addition, the discussion section explains the influence mechanisms of variables by combining details of the operation of local governments in Uganda.

8. The conclusion mentions “providing references for policymakers” but does not

propose specific plans based on key findings. Suggestion: Based on the model results, refine policy recommendations.

Thank you. Specific policy recommendations have been refined such as in Lines, 782-787. Additionally, recommendations for Statisticians or researchers have 837- 846.

9. The discussion of limitations only generally mentions that “indicator dimensions can be expanded and variables can be supplemented,” failing to address core deficiencies. The planning for future research directions is relatively vague and lacks specificity. Suggestion: Supplement the analysis of key limitations, clarify improvement paths, and demonstrate the sustainability of the research.

Thank you. A subsection on study limits has covered the known limitations, which mainly rotate around inadequate data. The planning for future studies in light of data limitations has been addressed in lines 837 – 846, for example the use of non-traditional data sources, use of more frequent data, etc.

10. For Figure 1, adding textual annotations for key steps to lower the comprehension barrier for readers.

Thank you. Narrative of Fig 1 has been included in Lines 334 to 343

11. Strengthen the correlation analysis between Figure 2 and Table 2. For example, supplement the discussion on “the impact of variables with high skewness/kurtosis on the distribution pattern of the composite indicator” and provide further explanations.

Thank you. A column has been added onto Table 2 to show that whereas many conceivable scenarios for computation of the CI were tested, the scores for only one approach were utilised based on the stability test. It is these figures that were used in Figure 2 to plot the distributions and further analyses.

12. For the “*” marking “Leptokurtic variable” in Table 2, clearly define the judgment criteria for kurtosis abnormalities in the table notes or main text.

Thank you. The judgement criteria have been included as a Table note but also in the text in Lines 520-522.

13. For the sub-figure titles in Figure 2, supplement explanations of the data period and analysis unit to enhance the self-explanatory nature of the figures

Thank you, the figure caption includes the data period and unit of analysis

14. There are grammatical issues in the sentences on Line 306 and Line 595; revisions are required. There are issues with the reference format. Please refer to the latest published papers and the requirements on the official website for correction.

Thank you. All grammatical errors and referencing styles have been addressed in line with the PLOS One requirements.

15. The paragraph format still needs adjustment, particularly regarding issues with first-line indentation

Thank you. This has been addressed throughout the paper.

16. Most of the literatures cited in the study are from 2021 or earlier, and relevant studies on service delivery of Ugandan local governments from 2022 to 2024 have not been included.

Thank you. Some of the accessed recent literature have been included such as 42, 40, 15 and 14.
